# A Novel Method to Improve the Anticancer Activity of Natural-Based Hydroxyapatite against the Liver Cancer Cell Line HepG2 Using Mesoporous Magnesia as a Micro-Carrier

**DOI:** 10.3390/molecules22121947

**Published:** 2017-11-24

**Authors:** Nasser S. Awwad, Ali M. Alshahrani, Kamel A. Saleh, Mohamed S. Hamdy

**Affiliations:** 1 Department of Chemistry, Science College, King Khalid University, P.O. Box 9004, Abha 61413, Saudi Arabia; nsawwad20@yahoo.com (N.S.A.); al_shahrani09@hotmail.com (A.M.A.); m.s.hamdy@gmail.com (M.S.H.); 2 Department of Biology, Science College, King Khalid University, P.O. Box 9004, Abha 61413, Saudi Arabia

**Keywords:** HAP@MgO, cytotoxicity, HepG2, liver cancer, micro-carriers

## Abstract

Micro-carriers are the best known vehicles to transport different kinds of drugs to achieve high impact. In this study, mesoporous magnesium oxide has been harnessed as a micro-carrier to encapsulate the anticancer candidate drug natural-based cubic hydroxyapatite (HAP). HAP@MgO composites with different HAP loading (0–60 wt %), were prepared by a hydrothermal treatment method using triethanol amine as a template. The characterization of the prepared composites were achieved by using XRD, Raman spectroscopy, FTIR and SEM. Characterization data confirm the formation of sphere-like structures of MgO containing HAP particles. It was observed that the size of the spheres increased with HAP loading up to 40 wt %, then collapsed. Furthermore, the anticancer property of the prepared composites was evaluated against the HepG2 liver cancer cell line. The HAP@MgO composites exhibited higher activity than neat MgO or HAP. The 20 wt % of HAP was the optimum loading to control cell proliferation by inducing apoptosis. Apoptosis was determined by typical apoptotic bodies produced by the cell membrane.

## 1. Introduction

Cancer is one of the major problems threatening public health around the world. Cancerous cells generally gain the ability to divide out of control of cell cycle check points leading to the formation of new cells characterized by uncontrolled growth, invasion and sometimes metastasis [1]. Liver; which is the organ responsible for detoxifying the body from different toxins, and therefore, it is the first target of poisons. This explains why liver cancer is considered as the second cause of death [2,3] compared to other cancer types. The main thought of several researchers is how to keep cancer cells under control, especially if it is not capable to remove the unwanted group of cells form the body without, or with low side effects. Unfortunately, the treatment methods which are used so far, like radiation and chemotherapy, have severe limitations [4]. Hence, it is necessary to seek alternative methods. One of the best new mechanisms is to harness the nano/micro-sized particles to attack or to carry the materials that can attack cancer cells only. In the literature, it has been reported that nano/micro-sized materials can be used for the detection and manipulation of different biological systems [5,6,7,8]. Formation of nanoparticles for medical purposes is one of the challenges that have attracted the attention of recent studies and investigations. This is due to their ability to be used in different applications like molecular diagnostics, drug and gene delivery vehicles, and cancer therapy [4].

Hydroxyapatite (HAP) is a calcium phosphate which is normally found in animal and human hard tissues [9]. Moreover, HAP has a hexagonal structure [10,11] with a stoichiometric Ca/P ratio of 1.67, [12,13]. Generally speaking, hydroxyapatite is a sustainable material that can be found easily in bio-waste such as animal bones, eggshells, and codfish bones [14,15,16]. The process of hydroxyapatite extraction from bio-waste implies total elimination of organic compounds present in bones [14], hence pure hydroxyapatite could be produced. Moreover, hydroxyapatite can be successfully prepared from cost-effective precursors such as calcite and urea phosphate [17]. The cytotoxic activity of hydroxyapatite against hepatocellular cancerous cells was investigated and reported by several research groups. Liu and coworkers [18] reported the use of synthetized hydroxyapatite on HepG2 cells. In another study, Li and coworkers [19] worked on the impact of nanoparticles as cytotoxic inducers of the activity of hepatocellular carcinoma cells. Moreover, Li and coworkers [20] reported the effect of uptake of synthetized nanoparticles such as rod-like hydroxyapatite into the hepatoma cells. Furthermore, in a very prestigious study, Cao and coworkers [21] reported the mechanism of inhibition of proliferation of hepatocellular carcinoma cells in unique in vitro and in vivo investigations.

Magnesium oxide (MgO), or the so called magnesia, is a stable inorganic material with some advantages; it is non-toxic and environmentally friendly [22,23]. The synthesis of mesoporous magnesia attracts researchers to improve its texture properties, i.e., surface area, diameter and volume the pores, rather than commercially available magnesia. Several methods were reported about the synthesis of mesoporous magnesia, such as sol-gel [24], hydrothermal decomposition [25], precipitation [26], chemical gas phase deposition [27], combustion aerosol process [28], and hard-templating techniques [29]. The ability of mesoporous magnesia to kill different cancerous cells such as AGS, SNU-16, and HeLa were explored [30]. Moreover, [31] reported the synthesis of mesoporous magnesia in a one-step hydrothermal synthesis procedure and the cytotoxic activity against hepatocellular (HepG2) cancer cells was investigated. Promising results were obtained and showed, not only significant impact on the HepG2 cell line, but also presented the ability to activate the mechanism of apoptotic pathways. The apoptotic pathway is known as the most acceptable way to control cancer cell death, because apoptotic cells are more capable to induce immune system [32,33].

In the current research, we attempt to increase the impact of natural-based hydroxyapatite obtained from camel bones by incorporating it with different loadings (0–60 wt %) into mesoporous magnesia. The prepared materials and their composites were characterized and the anticancer activity against HepG2 cells was investigated using the SulfoRhodamine-B (SRB) cytotoxicity method. Results showed that HAP@MgO composites exhibited higher activity against the liver cancer cell line compared to neat HAP or MgO separately. Moreover, the effect on apoptosis is reported.

## 2. Experimental

### 2.1. Synthesis

Natural-based hydroxyapatite was extracted from camel bones. The bones were cut to small pieces and immersed in diluted acetic acid for 24 h to remove fat and tissues. Then, the bones were washed several times using distilled water and dried at 220 °C for 48 h. After cooling, the bones were grinded to a very fine powder, then sieved to the particle size between 500 μm and 250 μm. Physical activation was carried out by carbonizing at 500 °C under N_2_ gas for 2 h, followed by activation under CO_2_ gas for 2 h at 600 °C.

The obtained hydroxyapatite was doped into mesoporous magnesia according the method adapted by Hamdy and coworkers [31]. Mesoporous magnesia was obtained by aging, drying, and calcining the homogeneous mixture of magnesium nitrate, triethanolamine (TEA) and tertraethyl ammonium hydroxide. Here, five samples of hydroxyapatite doped mesoporous magnesia were prepared with a loading of 0, 10, 20, 40, and 60 wt % of hydroxyapatite. In a typical synthesis, 15 g of TEA was diluted by 3.6 mL of H_2_O, and the resulting mixture was added dropwise to another slurry solution of magnesium nitrate 14.1 g/10 mL H_2_O in addition to the desired amount of hydroxyapatite while stirring. After stirring for about 30 min, 19.7 mL of tetraethyl ammonium hydroxide (TEAOH, 25%, Aldrich, Saint Louis, MO, USA) was added dropwise. Finally, the obtained colloidal solution was aged at room temperature for 24 h, dried at 100 °C for 24 h, heated in a Teflon-lined stainless steel autoclave at 180 °C for 4 h, and then calcined at 600 °C for 10 h by using a heating ramp rate of 1 degree/min under flow (30 mL/min) of 8% oxygen in nitrogen gas.

### 2.2. Characterization

The prepared composites were characterized using a Shimadzu LabX-XRD-6000 diffractometer with *CuK_α_* (*λ* = 1.5406 Å X-ray diffraction (XRD)), equipped with a digital library for pattern analysis. THERMO SCIENTIFIC DXR FT-Raman spectrometer was used to record Raman spectra with a laser source emitting at 532 nm and a power of 2 mW. Whereas THERMO SCIENTIFIC, DXR FT-IR spectrometer used to measure the FT-IR spectra of MgO and MgO@HAP by using KBr pellet method in the wavenumber range of 4000–400 cm^−1^. Finally, (FE-SEM) (JSM-7500 F; JEOL-Japan, Tokyo, Japan) scanning electron microscope was used to investigate the morphological structure of the prepared composites.

### 2.3. Cytotoxic Activity

In the present study, the SulphoRhodamine-B (SRB) method was chosen to detect the cytotoxicity of prepared samples and composites. Ethanol, methanol, and SRB stain were purchased from Sigma Chemical Co. (St. Louis, MO, USA). While Gibco/Life Technologies Co. (Carlsbad, CA, USA) products were used in cell culture like media and growth supplements. Other cell culture vessels were supplemented from Nunc Co. (Roskilde, Denmark).

Human hepatic carcinoma (HEPG-2) cells were from Vacsera (Giza, Egypt). HepG2 cells were maintained in cell culture media (RPMI) promoted with 100 units/mL penicillin streptomycin antibiotic. Two time weekly cells were sub-cultured in a humidified, 5% (*v*/*v*) CO_2_ atmosphere at 37 °C promoted with fetal bovine serum.

The anticancer activities of the prepared materials were tested against HepG-2 cells. Cells were cultured as different five groups: control non-treated cells, cells treated with Doxorubicin, cells treated with natural HAP, cells treated with MgO only, and cells treated with HAP@MgO composite. Collected results from our pre-experimental study revealed that the composite had the highest impact. Cells were exposed to different concentrations (10%, 20%, 40% and 60%) of the highest impact material, which was determine from the pre-experimental tests, in order to determine the most effective concentration. Cells were collected using 0.25% trypsin–EDTA and plated in 96-well plates. Cells were exposed to the prepared composites for 72 h and TCA (10%) was used to fix cells for 1 h at 4 °C. To remove TCA, cells were washed many times, then 0.4% SRB solution was used to stain cells in a dark place for 10 min. Subsequently, stained cells were washed with 1% glacial acetic acid. Finally, to dissolve SRB-stained cells, Tris–HCl was used. After drying overnight, the color intensity of remained cells was measured at 540 nm by Elisa.

For apoptotic body detection, cells were washed using PBS washing buffer twice and then collected using 0.25% trypsin–EDTA and transferred to staining slide. Cells were stained using ethidium bromide (EtB) and acridine Orange (AO) 1:1 concentration. Stained apoptotic bodies were detected and photographed under a Nikon Fluorescent microscope Japan. Finally, statistical analysis and IC_50_ calculation was performed using SigmaPlot version 12.0.

## 3. Results 

### 3.1. Material Characterization

**Figure 1:**
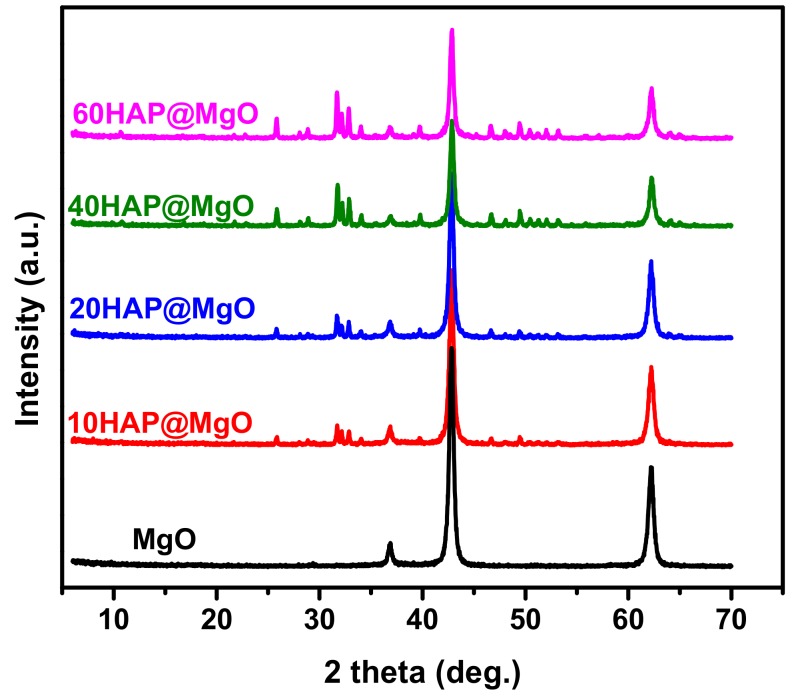
The X-Ray Diffraction (XRD) patterns of the prepared HAP@MgO composites as compared to the neat mesoporous MgO.

XRD patterns of the prepared composites are compared with those of mesoporous MgO in Figure 1. The neat mesoporous MgO pattern exhibits diffraction peaks at 36.8, 42.9 and 62.3, 2theta are characteristic of the face-centered cubic crystalline phase (periclase, JCPDS No. 45-0496) [33]. In the composites patterns, the distinguished peaks of MgO were found together with the peaks of HAP (JCPDS card No. 09-0432). It is clear that the intensity of MgO peaks are decreased with HAP loading, while the peaks of HAP are increased. More importantly, no peaks for other types of crystals were detected in the mesoporous MgO as an indication of the purity of the composites and the total removal of the template during the calcination step.

**Figure 2:**
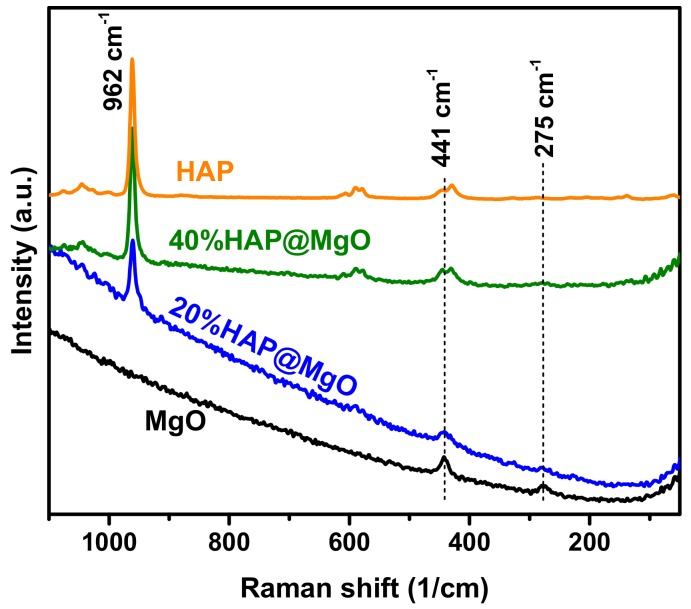
Raman spectra of the two references MgO and commercially available HAP, compared with that of the HAP@MgO composites.

Raman spectra of the prepared composites are compared with neat MgO and commercially available HAP in Figure 2. Neat MgO exhibited two bands at 275 and 441 cm^−1^. These two characteristics were also observed by Kim and coworkers [4], and they can be assigned to the TA phonon at the zone boundary and zone center, respectively [34,35]. The bands of MgO were also observed in the composites spectra; however, the intensities of the bands are decreased with HAP loading. In the composites spectra, a dominant band at 962 cm^−1^ was observed, which can be assigned to the internal modes of the PO_4_^3−^ tetrahedral *ν1* frequency corresponding to the symmetric stretching of P–O bonds. Moreover, two vibrational bands at 429 cm^−1^ (*ν2*) and 450 cm^−1^ (*ν2*) can be attributed to the O–P–O bending modes. The bands at 1046 cm^−1^ (*ν3*) and 1074 cm^−1^ (*ν3*) are assigned to the asymmetric *ν3* (P–O) stretching. Finally, the bands at 589 cm^−1^ and 608 cm^−1^ can be assigned to O–P–O bending character [36]. Hence, the composites exhibited the bands of MgO and the bands of HAP and no other bands were detected as an indication of the high purity of the prepared composites, and more importantly, no foreign phase could be detected. This result is consistent with XRD data.

**Figure 3:**
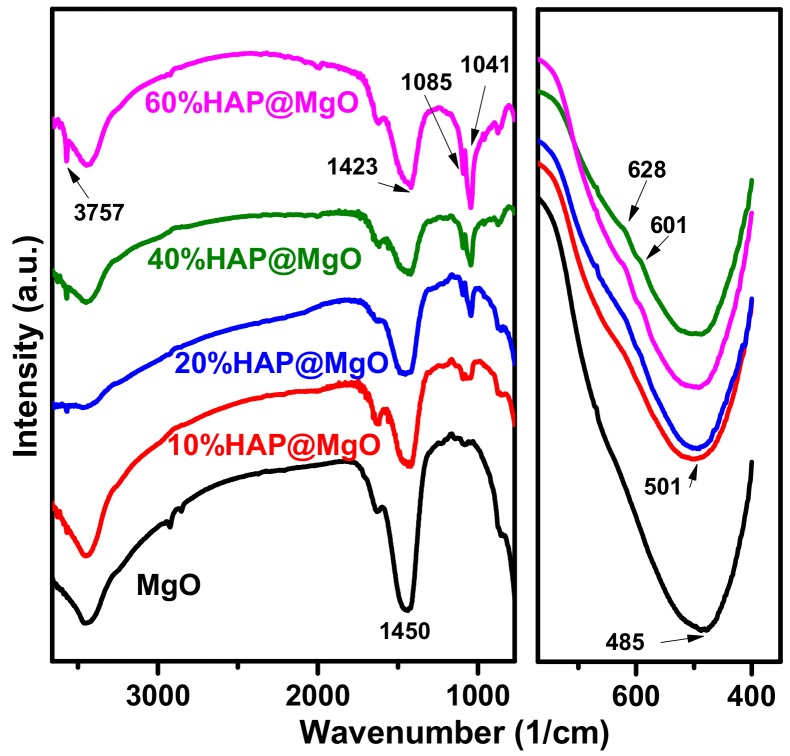
The FTIR spectra of the prepared HAP@MgO composites as compared to the neat mesoporous MgO.

Figure 3 shows the IR spectra of the HAP@MgO composites as compared with that of mesoporous MgO. Mesoporous MgO spectrum is dominated by three bands at 485, 1450 and 3420 cm^−^^1^, which can be attributed to the vibration mode of MgO, stretching vibration mode of the asymmetric COO^—^, and the OH stretching vibration of the adsorbed H_2_O molecules, respectively [34,36]. On the other hand, the vibrational modes of the PO_4_^−3^ group located at 601, 628, 1041, 1085, and 3757 cm^−1^ are developed with the HAP loading. The obtained IR band positions are matched with data reported by different research groups [37,38]. The IR data confirms the formation of HAP@MgO composites in a pure state without the presence of any other compound(s). The IR results are in agreement with XRD and Raman data.

**Figure 4:**
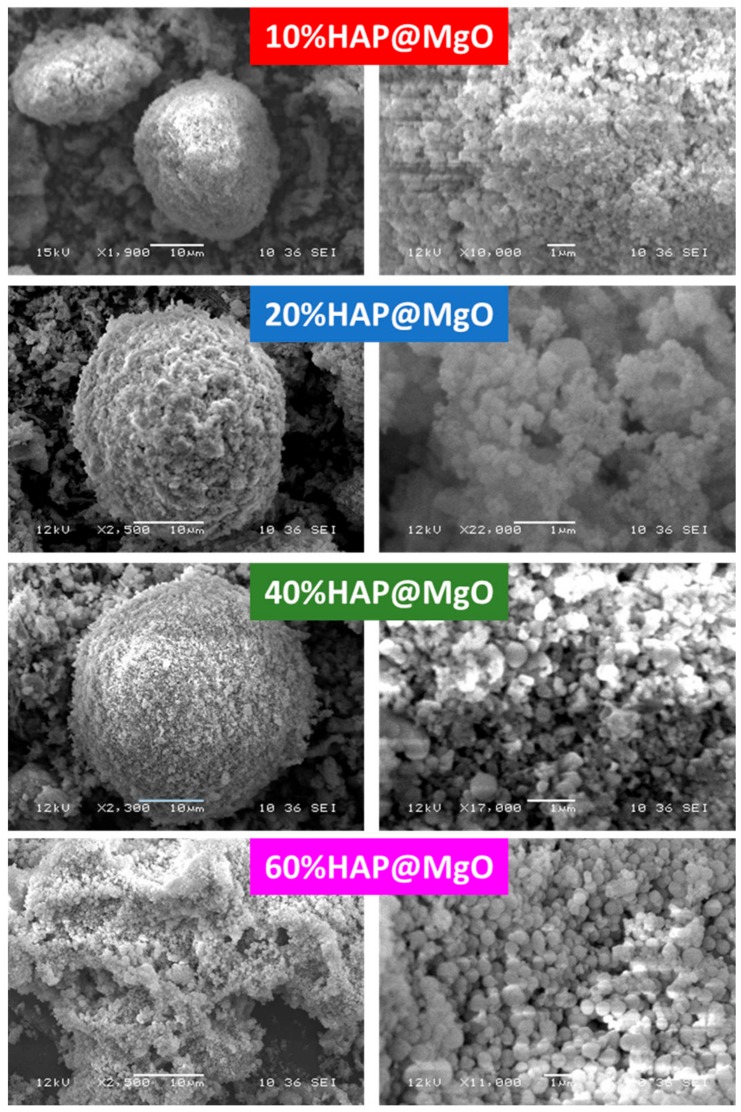
The Scanning Electron Microscope (SEM) micrographs of the prepared HAP@MgO composites.

**Figure 5:**
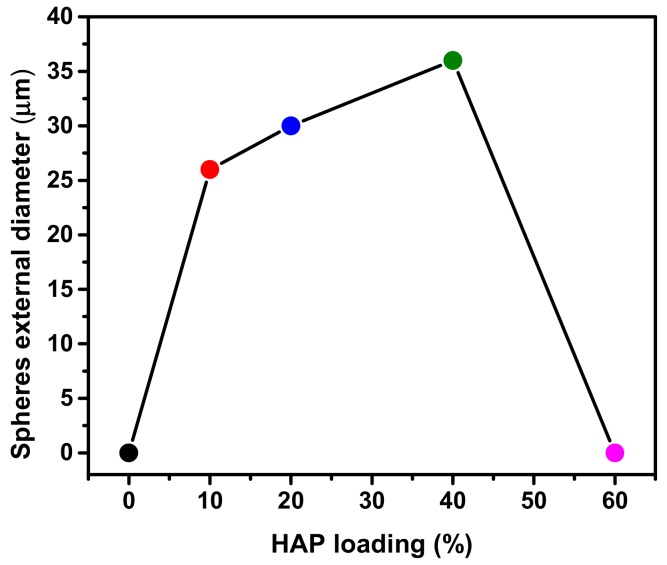
The external diameter of the sphere-like HAP@MgO as obtained from the SEM study as a function of HAP loading.

SEM micrographs of HAP@MgO composites are presented in Figure 4. The micrograph of 10HAP@MgO composite exhibited a sphere-like morphology with an external diameter of 26 µm, with a homogenous external surface of mainly MgO. The sphere-like morphology was observed also in the composites 20HAP@MgO and 40HAP@MgO, however, the diameter of the spheres increased to 30 and 36 µm, and the external surface seems to be heterogeneous where small cubic nano-crystals of HAP clearly appeared. The relationship between sphere size as a function of HAP loading can be seen in Figure 5. The sphere-like morphology was not maintained at high HAP loading. In 60HAP@MgO, the composite shows an irregular shape with an external surface made of mainly the cubic nano-crystals of HAP.

### 3.2. Cytotoxic Activity

**Figure 6:**
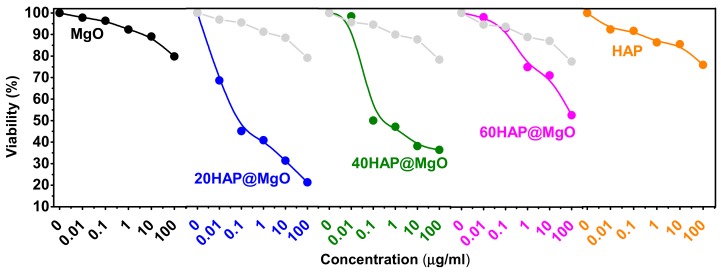
Dose response curves of HAP@MgO composites compared to that of neat MgO and HAP. Grey color curves represents the mathematical (theoretical) values of the composites activity.

Figure 6 presents the viability % of HepG2 cells after exposed to the prepared HAP@MgO composites, compared to the two references samples (neat MgO and neat HAP). Mesoporous MgO exhibited only 20.2% reduction in viability, while neat HAP showed 24.2% reduction. On the other hand, the HAP@MgO composites exhibited higher reduction. The composite 20HAP@MgO exhibited the highest reduction and almost 80% of the cells were poisoned, and 50% of cells present apoptotic bodies; 40HAP@MgO and 60HAP@MgO showed 66% and 48%, respectively. Moreover, the activity of the composites is different than the mathematical summation of the two neat samples of the composites (the gray curves in Figure 6). These results clearly confirm the synergy between HAP and MgO in the cytotoxic activity against HepG2 cells. The difference between the theoretical and the measured activity values follows the trend of 20HAP@MgO > 40HAP@MgO > 60HAP@MgO.

**Figure 7:**
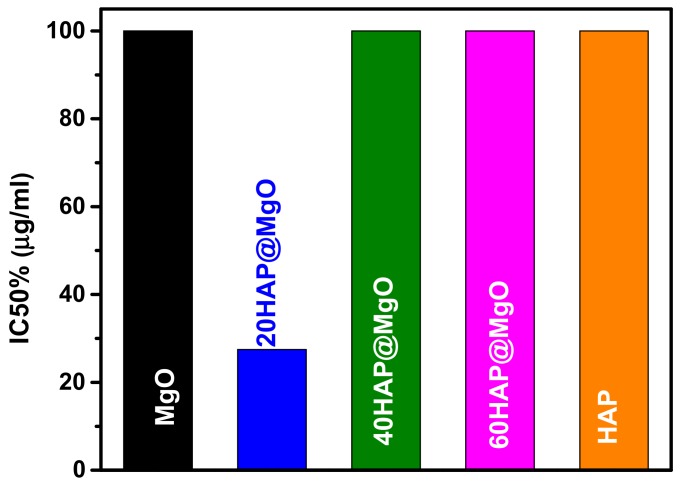
The IC_50_ values of the different composites against HepG2 cells by SulphoRhodamine-B (SRB) assay. HepG2 cells were exposed to various concentrations of HAP@MgO composites for 48 h.

The concentration of the composites where cancerous cells are reduced by half (IC_50_) is compared with that of the neat mesoporous MgO and HAP in Figure 7. The IC_50_ of the two reference samples, neat MgO and neat HAP, is >100 µg/mL. The composites, 40 and 60HAP@MgO showed IC_50_ >100 µg/mL. While, the composite 20 HAP@MgO was the best one with IC_50_ of 27.5 µg/mL.

## 4. Discussion

To the best of our knowledge, this is the first attempt to use MgO as a micro-carrier to facilitate the delivery of hydroxyapatite against HepG2 cancer cells. Kumar [39,40] showed the ability of bimetal ZnO–MgO to be a potential drug vehicle to carry doxorubicin against cancer cells [39]. However, one look at the MgO physical characteristics is sufficient to indicate that it is fit to be a potential carrier. Simply because, like other hydrophobic molecules, MgO will gain a bilayer spherical shape to avoid contact with water. The hydrophobic feature leads to a structure that looks like cell membranes. Accordingly, cells may take MgO by endocytosis, which means full delivery of charge. The present study confirmed that MgO alone has a significant IC_50_ impact. On the other hand, Ignjatovic [41] showed that coated hydroxyapatite compound can act as a selective anticancer drugs against lung cancer cells in vivo. This study aimed to increase the anticancer activity by producing of HAP@MgO composite. SRB stain results are consistent with what we had hoped to see. The comparison results between MgO and HAP@MgO composite show that the HAP@MgO composite increased the anticancer activity three times better than using MgO or HAP separately. This may reveal that MgO is a promising anticancer carrier.

To explore the mechanism of action of HAP@MgO composites, cells were stained with acridine orange and ethidium bromide mix (1:1). Interestingly, the anticancer activity of HAP@MgO composites showed a gradual decrease associated with increasing concentration of HAP. The highest effect was with 20HAP@MgO, while the effect gradually disappeared to be insignificant at 60HAP@MgO concentration. Both the impact and the mechanism of action were changed. While 20HAP@MgO showed a highly significant impact with weak apoptosis, 60HAP@MgO showed insignificant impact with weak necrosis. Some studies have explained that HAP can induce necrosis depending on the concentration of inducing TNF. In our opinion, this may be due to the ability of less concentration of HAP@MgO composite to pass cell membrane easily, while high concentration leads to accumulation of HAP@MgO outside the cell, which prevents the passage of oxygen molecules, so cells may undergo necrosis under the stress of oxygen deficiency, but not under induction of HAP@MgO.

**Figure 8:**
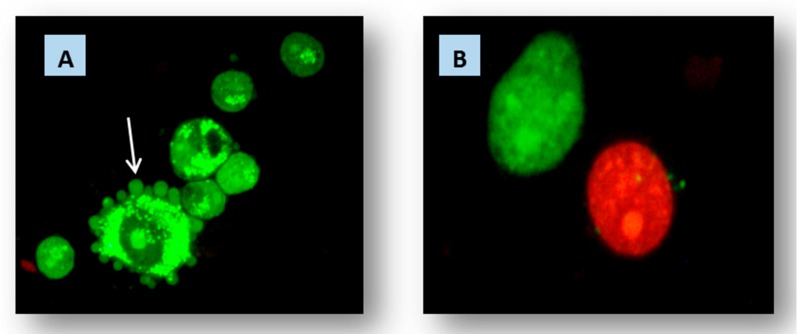
(**A**) HepG2 apoptotic bodies induced by 20HAP@MgO composite; (**B**) Necrosis (red cell) after treatment with 60HAP@MgO. Cells were incubated in a 5% CO_2_ incubator for 48 h. and stained with AO:Eth (1:1). Observation achieved using Olympus fluorescent microscope.

In addition to the previous observations and comments, results confirmed that the composites exhibit more cytotoxicity against HepG2 cells compared to their neat parent forms. The IC_50_ values have been given in Figure 7. Cytotoxicity is considered as a good anti-cancer parameter if it inducedes apoptotic pathways inside the cell. Apoptosis can be detected by many parameters like the activation of caspasees, DNA fragmentation, or changes to cell morphology. Depending on that, this study tried to identify if the composites can induce cells to undergo apoptosis or not by means of morphological parameters. The evaluation was based on nuclear shape, nuclear density, presence of foci, the number of cells, and presence of apoptotic bodies. Results clearly showed that nuclei retained their regular shape, did not condense, and no degradation of nuclei was detected (Figure 8A). Lack of micronuclei inside the cytoplasm revealed that carrier and composites are not clastogenic agents, which may be considered as very good feature and demonstrates MgO safety features confirmed before. Also, cells kept their ability to form foci, which means that the composites may have no impact on penetration of layers and have no ability to affect cell-to-cell connections. This can be considered as an advantage for the composites because they do not help cells to spread where they can be killed in situ. On the other hand, exposed cells clearly had apoptotic bodies. In terms of numbers of cells, photos have confirmed the compatibility with the SRB assay data. In light of previous results and discussion, HAP@MgO composites may be considered as in situ cytotoxic but not genototoxic. The optimum concentration of HAP that can induce apoptosis should not exceed 20% and should not be less than that. MgO is safe and has the ability to be a perfect micro-carrier candidate.

## 5. Conclusions

Natural-based nanoparticles of cubic hydroxyapatite were incorporated inside mesoporous magnesia by a hydrothermal treatment method. Composites with different HAP were characterized by several techniques which showed that MgO can accommodate the HAP nanoparticles up to 40% loading of HAP. The prepared HAP@MgO composites exhibited unique cytotoxic activity against HepG2 liver cancer cells. HAP@MgO composites may be considered as in situ cytotoxic, but not genototoxic agents. The optimum concentration of HAP that can induce apoptosis should not exceed 20%, and should not be less than that. MgO is safe and has the ability to be a micro-carrier candidate.

## References

[1] Almeida A.L., Martins J.B.L., Taft C.A., Longo E., Andres J., Lie S.K. (1998). A PM3 theoretical study of the adsorption and dissociation of water on MgO surfaces. J. Mol. Struct. THEOCHEM.

[2] Barakat N.A.M., Khil M.S., Omran A.M., Sheikh F.A., Kim H.Y. (2009). Extraction of pure natural hydroxyapatite from the bovine bones bio waste by three different methods. J. Mater. Process. Technol..

[3] Bian S.W., Baltrusaitis J., Galhotra P., Grassian V.H. (2010). A template-free, thermal decomposition method to synthesize mesoporous MgO with a nanocrystalline framework and its application in carbon dioxide adsorption. J. Mater. Chem..

[4] Bouyer E., Gitzhofer F., Boulos M.I. (2000). Morphological study of hydroxyapatite nanocrystal suspension. J. Mater. Sci. Mater. Med..

[5] Buzea C., Pacheco I.I., Robbie K. (2007). Nanomaterials and nanoparticles: Sources and toxicity. Biointerphases.

[6] Castelli G., Pelosi E., Testa U. (2017). Liver Cancer: Molecular Characterization, Clonal Evolution and Cancer Stem Cells. Cancers.

[7] Castillo J., Dimaki M., Svendsen W.E. (2009). Manipulation of biological samples using micro and nano techniques. Integr. Biol..

[8] Chalkidou A.S., Boutis A.L., Padelis P. (2009). Management of a Solitary Bone Metastasis to the Tibia from Colorectal Cancer. Case Rep. Gastroenterol..

[9] Ciobanu C.S., Iconaru S.L., Massuyeau F., Constantin L.V., Costescu A., Predoi D. (2012). Synthesis, Structure, and Luminescent Properties of Europium-Doped Hydroxyapatite Nanocrystalline Powders. J. Nanomater..

[10] Cui H., Wu X., Chen Y., Boughton R.I. (2014). Synthesis and characterization of mesoporous MgO by template-free hydrothermal method. Mater. Res. Bull..

[11] Etacheri V., Roshan R., Kumar V. (2012). Mg-doped ZnO nanoparticles for efficient sunlight-driven photocatalysis. ACS Appl. Mater. Interfaces.

[12] Falah R.R., Talib W.H., Shbailat S.J. (2017). Combination of metformin and curcumin targets breast cancer in mice by angiogenesis inhibition, immune system modulation and induction of p53 independent apoptosis. Ther. Adv. Med. Oncol..

[13] Ferrer-Miralles N., Rodriguez-Carmona E., Corchero J.L., Garcia-Fruitos E., Vazquez E., Villaverde A. (2015). Engineering protein self-assembling in protein-based nanomedicines for drug delivery and gene therapy. Crit. Rev. Biotechnol..

[14] Guo L., Huang M., Zhang X. (2003). Effects of sintering temperature on structure of hydroxyapatite studied with Rietveld method. J. Mater. Sci. Mater. Med..

[15] Ha S.W., Jang H.L., Nam K.T., Beck G.R. (2015). Nano-hydroxyapatite modulates osteoblast lineage commitment by stimulation of DNA methylation and regulation of gene expression. Biomaterials.

[16] Hamdy M.S., Awwad N.S., Alshahrani A.M. (2016). Mesoporous magnesia: Synthesis, characterization, adsorption behavior and cytotoxic activity. Mater. Des..

[17] Han Y., Li S., Cao X., Yuan L., Wang Y., Yin Y., Qiu T., Dai H., Wang X. (2014). Different inhibitory effect and mechanism of hydroxyapatite nanoparticles on normal cells and cancer cells in vitro and in vivo. Sci. Rep..

[18] Ibrahim A.R., Wei W., Zhang D., Wang H., Li J. (2013). Conversion of waste eggshells to mesoporous hydroxyapatite nanoparticles with high surface area. Mater. Lett..

[19] Ignjatović N.L., Penov-Gaši K.M., Wu V.M., Ajduković J.J., Kojić V.V., Vasiljević-Radović D., Kuzmanović M., Uskoković V., Uskoković D.P. (2016). Selective anticancer activity of hydroxyapatite/chitosan-poly(d,l)-lactide-co-glycolide particles loaded with an androstane-based cancer inhibitor. Colloids Surf. B Biointerfaces.

[20] Ishikawa K., Fujima N., Komura H. (1985). First-order Raman scattering in MgO microcrystals. J. Appl. Phys..

[21] Kim H.S., Kim H.W. (2009). Fabrication and Raman Studies of MgO/SnO_2_ Core-Shell Hetero-nanowires. Acta Phys. Pol..

[22] Krischok S., Stracke P., Höfft O., Kempter V., Zhukovskii Y.F., Kotomin E.A. (2006). A comparative analysis of electron spectroscopy and first-principles studies on Cu(Pd) adsorption on MgO. Surf. Sci..

[23] Kumar R., Gokulakrishnan N., Kumar R., Krishna V.M., Saravanan A., Supriya S., Somanathan T. (2015). Can Be a Bimetal Oxide ZnO-MgO Nanoparticles Anticancer Drug Carrier and Deliver? Doxorubicin Adsorption/Release Study. J. Nanosci. Nanotechnol..

[24] Kumaran R.S., Choi Y.K., Singh V., Song H.J., Song K.G., Kim K.J., Kim H.J. (2015). In vitro cytotoxic evaluation of MgO nanoparticles and their effect on the expression of ROS genes. Int. J. Mol. Sci..

[25] Lazaridis N.K., Kyzas G.Z., Vassiliou A.A., Bikiaris D.N. (2007). Chitosan Derivatives as Biosorbents for Basic Dyes. Langmuir.

[26] Li J., Li Y., Zhang L., Zuo Y. (2008). Composition of calcium deficient Na-containing carbonate hydroxyapatite modified with Cu(II) and Zn(II) ions. Appl. Surf. Sci..

[27] Li W.C., Lu A., Weidenthaler C., Schüth F. (2004). Hard-Templating Pathway To Create Mesoporous Magnesium Oxide. Chem. Mater..

[28] Lo W.J., Grant D.M., Ball M.D., Welsh B.S., Howdle S.M., Antonov E.N., Bagratashvili V.N., Popov V.K. (2000). Physical, chemical, and biological characterization of pulsed laser deposited and plasma sputtered hydroxyapatite thin films on titanium alloy. J. Biomed. Mater. Res..

[29] Maffei A.V., Budd P.M., McKeown N.B. (2006). Adsorption Studies of a Microporous Phthalocyanine Network Polymer. Langmuir.

[30] Mahmoud A., Ezgi O., Merve A., Ozhan G. (2016). In Vitro Toxicological Assessment of Magnesium Oxide Nanoparticle Exposure in Several Mammalian Cell Types. Int. J. Toxicol..

[31] Piccirillo C., Silva M.F., Pullar R.C., da Cruz I.B., Jorge R., Pintado M.M.E., Castro P.M.L. (2013). Extraction and characterisation of apatite- and tricalcium phosphate-based materials from cod fish bones. Mater. Sci. Eng. C.

[32] Reyes-Gasga J., Martinez-Pineiro E.L., Bres E.F. (2012). Crystallographic structure of human tooth enamel by electron microscopy and X-ray diffraction: Hexagonal or monoclinic?. J. Microsc..

[33] Rogina A., Ivanković M., Ivanković H. (2013). Preparation and characterization of nano-hydroxyapatite within chitosan matrix. Mater. Sci. Eng. C.

[34] Shen Y., Ahmad M.R., Nakajima M., Kojima S., Homma M., Fukuda T. (2011). Evaluation of the single yeast cell’s adhesion to ITO substrates with various surface energies via ESEM nanorobotic manipulation system. IEEE Trans. Nanobiosci..

[35] Siegel R.L., Miller K.D., Jemal A. (2015). Cancer statistics, 2015. CA Cancer J. Clin..

[36] Stark J.V., Klabunde K.J. (1996). Nanoscale Metal Oxide Particles/Clusters as Chemical Reagents. Adsorption of Hydrogen Halides, Nitric Oxide, and Sulfur Trioxide on Magnesium Oxide Nanocrystals and Compared with Microcrystals. Chem. Mater..

[37] Vu A.T., Jiang S., Ho K., Lee J.B., Lee C. (2015). Mesoporous magnesium oxide and its composites: Preparation, characterization, and removal of 2-chloroethyl ethyl sulfide. Chem. Eng. J..

[38] Yin M., Yin Y., Han Y., Dai H., Li S. (2014). Effects of Uptake of Hydroxyapatite Nanoparticles into Hepatoma Cells on Cell Adhesion and Proliferation. J. Nanomater..

[39] Yin M.Z., Han Y.C., Bauer I.W., Chen P., Li S.P. (2006). Effect of hydroxyapatite nanoparticles on the ultrastructure and function of hepatocellular carcinoma cells in vitro. Biomed. Mater..

[40] Yuan Y., Liu C., Qian J., Wang J., Zhang Y. (2010). Size-mediated cytotoxicity and apoptosis of hydroxyapatite nanoparticles in human hepatoma HepG2 cells. Biomaterials.

[41] Zhou J., Yang S., Yu J. (2011). Facile fabrication of mesoporous MgO microspheres and their enhanced adsorption performance for phosphate from aqueous solutions. Colloids Surf. A Physicochem. Eng. Asp..

